# Correction to: Privacy-aware estimation of relatedness in admixed populations

**DOI:** 10.1093/bib/bbae099

**Published:** 2024-02-28

This is a correction to:

Su Wang, Miran Kim, Wentao Li, Xiaoqian Jiang, Han Chen, Arif Harmanci, Privacy-aware estimation of relatedness in admixed populations, *Briefings in Bioinformatics*, Volume 23, Issue 6, November 2022, bbac473, https://doi.org/10.1093/bib/bbac473

In the originally published version of this manuscript, author affiliations are not presented explicitly in the pdf version of the paper.

The author affiliations should be represented in the following way within the PDF document:

Su Wang and Wentao Li are graduate students working in genomics and privacy. Their current affiliation is The University of Texas Health Science Center Houston, D. Bradley McWilliams School of Biomedical Informatics.

Miran Kim is an expert in genomic data security, cryptography, privacy, and algorithm development. Dr. Kim's current affiliation is Hanyang University, Department of Mathematics, Seoul, Republic of Korea.

Han Chen has broad expertise in statistical genetics. Dr. Chen's current affiliation is The University of Texas Health Science Center Houston, School of Public Health.

Arif Harmanci and Xiaoqian Jiang are researchers with focus on genomics, privacy, and AI/ML methods. Their current affiliation is The University of Texas Health Science Center Houston, D. Bradley McWilliams School of Biomedical Informatics.

These details have been corrected only in this correction notice to preserve the published version of record.

